# Antileishmanial and antitrypanosomal activity of the cutaneous secretion of *Siphonops annulatus*

**DOI:** 10.1186/1678-9199-20-50

**Published:** 2014-11-24

**Authors:** Erika Gracielle Pinto, Marta Maria Antoniazzi, Carlos Jared, Andre Gustavo Tempone

**Affiliations:** Departamento de Parasitologia e Micologia, Instituto Adolfo Lutz, Av. Dr. Arnaldo, 351, 8° andar, CEP 01246-000 São Paulo, SP Brasil; São Paulo Institute of Tropical Medicine, University of São Paulo (USP), São Paulo, São Paulo State Brazil; Laboratory of Cell Biology, Butantan Institute, São Paulo, São Paulo State Brazil

**Keywords:** Amphibians, Venoms, *Leishmania*, *Trypanosoma cruzi*, Therapy, Drugs

## Abstract

**Background:**

Among the tropical parasitic diseases, those caused by protozoans are considered a challenge to public health, being represented by leishmaniasis and Chagas disease. In view of the low effectiveness and toxicity of the current therapy, animal venoms such as amphibian secretions have been used as a promising source of new drug prototypes. The present work aimed to achieve bioguided fractionation of metabolites present in a cutaneous secretion of the caecilian *Siphonops annulatus* (Amphibia: Gymnophiona: Siphonopidae) with antileishmanial and antitrypanosomal activity.

**Methods:**

Through liquid-liquid partition and chromatographic techniques, the secretion was fractionated using bioguided assays. The 50% inhibitory concentration (IC_50_) of the main fraction (SaFr1) was studied against *Leishmania* (L.) *infantum* promastigotes and intracellular amastigotes, trypomastigotes of *Trypanosoma cruzi* and mammalian cells; viability was detected by the colorimetric MTT assay. By using a spectrofluorimetric assay with the probe SYTOX® Green and transmission electron microscopy (TEM), we also investigated the potential damage caused by SaFr1 in the plasma membrane and mitochondria of *Leishmania*.

**Results:**

The bioguided assay enabled isolation of a highly purified fraction (SaFr1) with an IC_50_ of 0.065 μg/mL against promastigotes and 2.75 μg/mL against trypomastigotes. Due to its high toxicity to peritoneal macrophages, SaFr1 showed no selectivity towards the intracellular forms of *Leishmania*. Ultrastructural studies with *Leishmania* demonstrated severe mitochondrial damage and the formation of large cytoplasmic vacuoles, leading to the parasite’s death within a few hours. Nevertheless, it caused no alteration in the plasma membrane permeability as detected by the fluorescent probe and TEM.

**Conclusions:**

The present study demonstrated for the first time the antiparasitic activity of the skin secretion of the caecilian *S. annulatus* against *Leishmania* and *T. cruzi*, confirming that skin secretions of these amphibians, similarly to those of anurans and salamanders, are also potential tools for the development of new drug candidates against neglected diseases.

## Background

Among the natural toxins used as important tools in the development of new drugs, amphibian secretions represent a rich source of metabolites, providing potential candidates for drug design studies. Leishmaniasis is a complex of diseases caused by protozoan parasites of the genus *Leishmania*. Included among the tropical neglected diseases, it is endemic in 98 countries, with a global incidence estimated at approximately 1.3 million cases each year; Brazil is included among the ten countries with the highest number of estimated cases [1]. The therapy is based on the use of a few drugs such as pentavalent antimonials and amphotericin B, with medium to severe toxic side effects and long-term administration [2].

American trypanosomiasis or Chagas disease has been considered among the most neglected afflictions in several underdeveloped and developing countries, and affects about 28 million people in Latin America [3]. It is caused by the flagellate protozoan parasite *Trypanosoma cruzi* and is transmitted to humans by triatomine insects primarily during insect feeding, or by blood transfusion and congenital transmission [4]. The only drug available in Brazil within the highly limited therapeutic arsenal is benznidazole, which presents limited efficacy in the chronic phase and high toxicity [5]. Thus, there is an essential need for the discovery of new drug entities, using animal toxins as a source [6, 7].

Amphibian secretions represent a rich source of bioactive peptides, including metabolites such as steroids, alkaloids, biogenic amines and proteins [8–10]. A variety of antimicrobial peptides (AMPs) present in amphibian skin secretion has been well characterized, showing activity against bacteria, such protozoan parasites as *Trypanosoma cruzi* and *Leishmania*, fungi and viruses [8, 11–15].

In the present work, by using bioguided fractionation, we investigated the antiprotozoal activity of metabolites isolated from the cutaneous secretion of the Brazilian caecilian *Siphonops annulatus* (Amphibia: Gymnophiona: Siphonopidae) using liquid-liquid partition and reversed-phase ultra-performance liquid chromatography, coupled to a photodiode array detector (RP-UPLC-PDA). We also investigated the ultrastructural damage in *Leishmania*, as well as the potential interference of the active fraction in the plasma membrane permeability of the parasite, and its mammalian cytotoxicity.

## Methods

### Materials

Sodium dodecyl sulfate (SDS), 3-[4,5-dimethylthiazol-2-yl]-2,5-diphenyltetrazolium bromide (MTT; thiazol blue), M-199 and RPMI-PR-1640 medium were purchased from Sigma (St. Louis, USA). Pentavalent antimony (Glucantime®, Aventis-Pharma, Brazil) miltefosine and benznidazole were used as standard drugs. All solvents used were grade HPLC of J.T. Baker. The other analytical reagents were purchased from Sigma (St. Louis, USA).

### Amphibian secretion

Specimens of *Siphonops annulatus* were collected at Ilhéus (Bahia state, Brazil). The skin secretion of *S. annulatus* (800 mg) was obtained by mild mechanical stimulus of animals submerged in deionized water at Butantan Institute. The secretion was then lyophilized and stored at −20°C.

### Bioguided amphibian-secretion fractionation

The pre-fractionation of crude venom was conducted using liquid-liquid partition with solvents of increasing polarity (n-hexane, ethyl acetate and butane). The hexane fraction (SaFrHex) and ethyl acetate fraction of *S. annulatus* (SaFrAcEt) were analyzed by reversed-phase ultra-performance liquid chromatography (RP-UPLC) using a binary UPLC system (20A Prominence, Shimadzu Co., Japan), in a C18 column (ACE C18, 5 μm, 100 Å, 250 mm × 4.6 mm). These fractions were diluted in methanol and applied in the column (10 μL). The column was eluted at a constant flow rate of 1 mL.min^−1^ in a gradient of methanol (solvent B) and water (solvent A). Elution of the fractions were monitored in a range of 200–800 nm, using a photodiode array detector. The fractions with the same retention time were pooled. The biological activity was detected using *in vitro* incubation of fractions of *L. (L.) infantum* promastigotes and *Trypanosoma cruzi* trypomastigotes for 24 hours.

### Bioassay procedures

BALB/c mice and golden hamsters were supplied by the Animal Breeding Facility at the Adolfo Lutz Institute of São Paulo. They were maintained in sterilized cages under a controlled environment, receiving water and food *ad libitum*. Animal procedures were performed with the approval of the Research Ethics Commission, in agreement with the Guide for the Care and Use of Laboratory Animals from the National Academy of Sciences (http://www.nas.edu).

### Parasite maintenance

*L. (L.) infantum* (MHOM/BR/1972/LD) was maintained in Golden hamsters, up to approximately 60 to 70 days post-infection [16]. Promastigotes were maintained in M-199 medium supplemented with 10% calf serum and 0.25% hemin at 24°C. Trypomastigotes of *Trypanosoma cruzi* were maintained in LLC-MK2 (ATCC CCL 7) cells using Roswell Park Memorial Institute (RPMI) 1640 medium supplemented with 2% calf serum at 37°C in a 5% CO_2_ incubator [17].

### Mammalian cells

Peritoneal macrophages were collected from the peritoneal cavity of female BALB/c mice by washing with RPMI-1640 without phenol red, supplemented with 10% fetal calf serum. Rhesus monkey kidney cells (LLC-MK2) were maintained in RPMI-1640 medium without phenol red and supplemented with 10% fetal calf serum at 37°C in a 5% CO_2_ incubator [17].

### Determination of the 50% inhibitory concentration (IC_50_)

#### Antileishmanial activity

##### Promastigotes

The antileishmanial activity was determined in *L. (L.) infantum* promastigotes, which were counted in a Neubauer hemocytometer and seeded at 1 × 10^6^ cells per well in 96-well microplates using miltefosine as the standard drug. The SaFrHex and SaFrAcEt were tested at 300 μg/mL (based on dry weight). The active fraction (SaFr1) was tested for 24 hours at 24°C, in a concentration range between 150 μg/mL and 0.02 μg/mL (based in dry weight), using a serial dilution (base 2). The parasite viability was determined using the MTT assay at 570 nm [18]. Briefly, 3-[4,5-dimethylthiazol-2-yl]-2,5-diphenyltetrazolium bromide (MTT) was dissolved in phosphate-buffered saline (PBS) at 5 mg/mL and incubated with cells (20 μL/well) for four hours under the same conditions. The extraction of the mitochondrial formazan was done with 80 μL of 10% SDS, followed by 24-hour incubation at 24°C. The optical density was read at 570 nm (Multiskan) using control wells with *Leishmania* and without drugs (100% viability), with 0.5% methanol, and without parasites (blank wells).

##### Amastigotes

Briefly, peritoneal macrophages were seeded at 1 × 10^5^ cells/well in 16-well slide chambers (NUNC®) (Thermo Scientific™, USA), infected with the previously isolated (hamster spleen) *L. (L.) infantum* amastigotes in a 1:10 ratio (macrophage/amastigote) and treated with drugs for 120 hours at 37°C in an incubator humidified with 5% CO_2_. At the end of the assay, the macrophages were fixed with methanol, stained with Giemsa and observed using light microscopy. The parasite burden was verified using the number of infected macrophages out of 400 cells [19] and Glucantime® was used as the standard drug. The data obtained represent the mean of two independent assays, and each assay was performed in duplicate.

#### Antitrypanosomal activity

Free trypomastigotes obtained from LLC-MK2 cultures were counted in a Neubauer hemocytometer and seeded at 1 × 10^6^ cells per well in 96-well microplates. The active fraction (SaFr1) was incubated at 150 μg/mL for 24 hours at 37°C in a 5% CO_2_ incubator. Benznidazole was used as the standard drug. Parasite viability was determined using the MTT assay, as previously described [20].

### Cytotoxicity against mammalian cells

Macrophages were obtained as previously described and seeded at 4 × 10^5^ cells per well in 96-well microplates. The cells were incubated with the active fraction (SaFr1) at 100 μg/mL for 48 hours at 37°C in a 5% CO_2_ incubator. The viability of macrophages was determined using the MTT assay as previously described [18]. Miltefosine, Glucantime® and benznidazole were used as standard drugs [8].

### Transmission electron microscopy analysis

*L. (L.) infantum* promastigotes were incubated with *S. annulatus* active fraction (SaFr1) and the ultrastructural changes were observed for different periods (1, 2, 4, 16 hours). Subsequently, promastigotes were fixed in 2.5% glutaraldehyde in 0.1 M sodium cacodylate/0.2 M sucrose buffer, pH 7.2. One percent osmium tetroxide post-fixation was followed by 1% uranyl acetate treatment. The samples were dehydrated through an acetone series and embedded in Epon. Ultrathin sections were stained with uranyl acetate and lead citrate. The material was examined in a JEOL 1011 transmission electron microscope (USA).

### Evaluation of the *Leishmania*plasma membrane permeability

Late-growth-phase (non-stationary) promastigotes were washed in PBS, seeded at 2 × 10^6^/well and incubated with 1 μM SYTOX® Green (Molecular Probes®, Invitrogen, USA) for 15 minutes at 24°C, in the dark [21]. The active fraction (SaFr1) was added and the fluorescence was measured every 30 minutes (up to 120 minutes). The control with maximum permeabilization was obtained with 0.1% Triton X-100. The fluorescence intensity was determined using a fluorimetric microplate reader (FilterMax™ F5 Multi-Mode Microplate Reader, Molecular Devices, USA) with excitation and emission wavelengths of 485 and 520 nm, respectively.

### Statistical analysis

The obtained data represent the mean and standard deviation of duplicate samples from two independent assays. The IC_50_ values were calculated using sigmoid dose–response curves generated by the software GraphPad Prism version 5.0 for Windows (GraphPad Software, USA), and the 95% confidence intervals are included in parentheses.

## Results

### *S. annulatus*secretion fractionation

**Figure 1:**
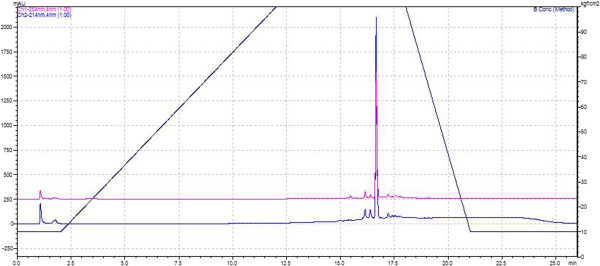
**Chromatographic profile of hexane and ethyl acetate unified fractions obtained by RP-UPLC-PDA in a C18 column (ACE C18, 5 μm, 100 Å, 250 mm × 4.6 mm) at a constant flow rate of 1 mL/min** ^**−1**^ **using water as solvent A and MeOH as solvent B.** The chromatogram was obtained at wavelengths of 214 and 254 nm.

The HPLC-bioguided fractionation of the *S. annulatus* secretion showed two active fractions (n-hexane and ethyl acetate). Analytical chromatography revealed the presence of a major peak in both fractions. After comparing their retention times and the similarity of the chromatographic profile of the hexane and ethyl acetate fractions, the peaks with the same retention times were pooled (named SaFr1), resulting in a high purity fraction (98%) as determined by RP-UPLC-PDA (Figure 1). After several injections, the total amount of SaFr1 isolated was 1.3 mg, which comprised only 0.16% of the total mass.

### Antiparasitic activity and mammalian toxicity

The hexane and ethyl acetate fractions eliminated 100% of promastigotes at the tested concentrations. Promastigotes were incubated with SaFr1 for 24 hours at different concentrations and the cell viability was determined by the MTT method. The SaFr1 was active at the highest tested concentration, with an IC_50_ value of 0.065 μg/mL (95% CI 0.0519 to 0.0816 μg/mL). Miltefosine was used as a standard drug and showed an IC_50_ value of 27.26 μg/mL (95% CI 25.63 to 28.97 μg/mL) (Table 1). Subsequently, the SaFr1 was tested against the intracellular *Leishmania* amastigotes, but it showed no activity.

**Table 1:** **Antiparasitic activity and cytotoxicity of SaFr1, the viability was determined by the colorimetric assay of MTT at 570 nm**

Compounds	IC _50_ mμg/mL) (95% CI)			
	***L. (L.) infantum*** promastigotes	***L. (L.) infantum*** amastigotes	***T. cruzi*** trypomastigotes	Macrophages cytotoxicity
SaFr1	0.065 (0.051-0.081)	NA	2.755 (2.235-3.396)	0.278 (0.231-0.336)
Miltefosine	27.26 (25.63-28.97)	17.80 (11.57-24.57)	–	122.0 (94.78-157.00)
Glucantime	NA	25.0 (24.40-25.63)	–	>200
Benznidazole	–	–	114.68 (105.69-124.49)	469.93 (414.98-532.18)

IC_50_: 50% inhibitory concentration; 95% CI: 95% confidence interval; NA: not active.

Trypomastigotes of *T. cruzi* were incubated with SaFr1 for 24 hours at different concentrations and the cell viability was also determined by MTT assay. The SaFr1 showed an IC_50_ value of 2.75 μg/mL (95% CI 2.235 to 3.396 μg/mL) (Table 1). Benznidazole was used as the standard drug and showed an IC_50_ value of 114.68 μg/mL (95% CI 105.69 to 124.49 μg/mL).

In order to evaluate the cytotoxicity to mammalian cells, peritoneal macrophages were incubated for 48 hours with SaFr1 and the cell viability was determined by the MTT method. The SaFr1 showed an IC_50_ value of 0.278 μg/mL (95% CI 0.231 to 0.336 μg/mL) (Table 1).

### Ultrastructural studies

**Figure 2:**
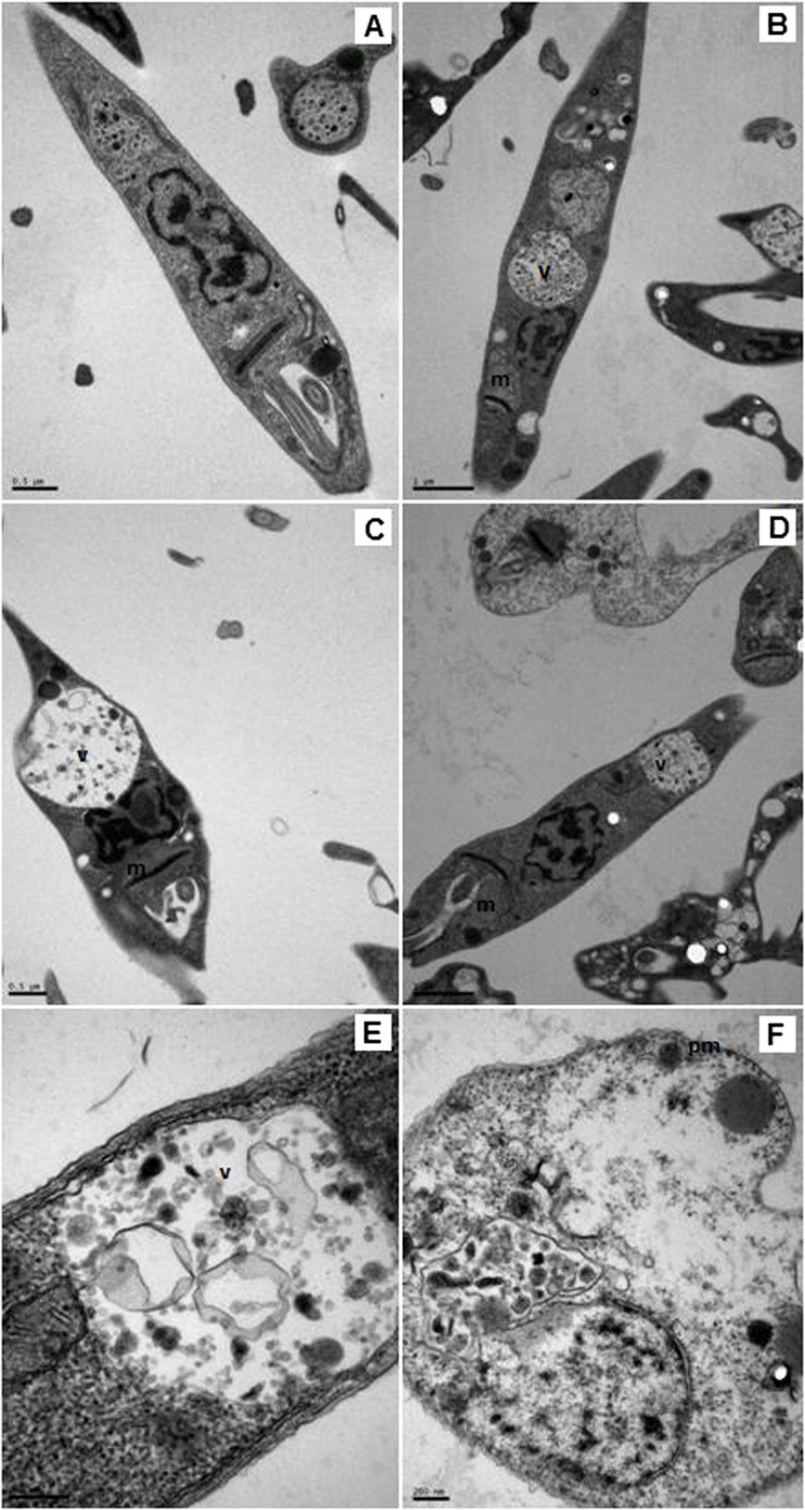
**Evaluation of ultrastructural damage by transmission electron microscopy (TEM).** Promastigotes of *L. (L.) infantum* were incubated with SaFr1 at different times: **(A)** control (untreated); **(B)** one-hour incubation; **(C)** two-hour incubation; **(D)** and **(E)** four-hour incubation and **(F)** 16-hour incubation. m: mitochondrial damage; v: vacuole; pm: plasma membrane integrity. Bar represents **(A)** 0.5 μm; **(B)** 1 μm; **(C)** 0.1 μm; **(D)** 0.5 μm; **(E)** 100 nm; **(F)** 100 nm.

The ultrastructural studies of *L. (L.) infantum* promastigotes demonstrated an intense time-dependent cellular damage, namely mitochondrial swelling within the initial incubation times (1–4 hours), and loss of cytoplasmatic organelles at 16 hours, leading to parasite death (Figure 2). At one hour of incubation (Figure 2B), a pronounced swelling of mitochondria and an increasing formation of large cytoplasmic vacuoles were observed; after two hours of incubation (Figure 2C), the morphology of the parasite became visibly altered. At four hours of incubation (Figures 2D and E), there was a continuous mitochondrial damage and formation of vacuoles containing cellular membranes. Finally, after 16 hours of incubation (Figure 2F), all cytoplasmic organelles were lost, including nucleoli, with a predominant round shape, confirming the death of the parasite. Although the SaFr1 caused intense cellular damage, no pore-forming activity was observable in the plasma membrane, which showed a preserved microtubule architecture.

### Evaluation of the plasma membrane permeability of *L. (L.) infantum*promastigotes

**Figure 3:**
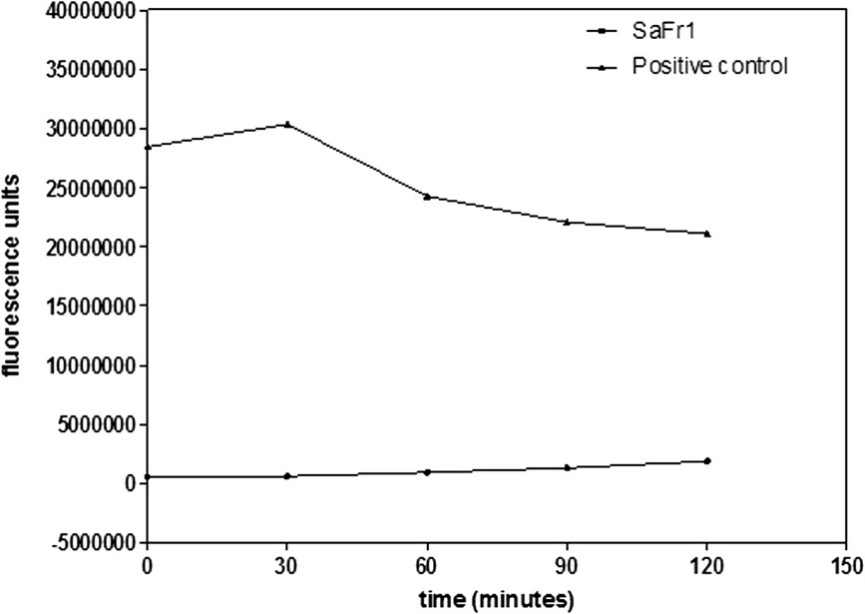
**Evaluation of the plasma membrane permeability of** ***L. (L.) infantum*** **incubated with SaFr1 by SYTOX Green®.** The fluorescence intensity was determined using a fluorimetric microplate reader with excitation and emission wavelengths of 485 and 520 nm, respectively. Triton X-100 was used as the positive control of the assay.

The SaFr1 was incubated with promastigotes and the plasma membrane permeability was evaluated with vital dye SYTOX® Green. The SaFr1 showed no interference with the plasma membrane after 120 minutes of incubation, which signifies no increase in the fluorescence intensity when compared to the untreated control group. Triton-X 100 was used as a positive control and resulted in an elevated fluorescence intensity (Figure 3).

## Discussion

Amphibian secretions have been used as an important tool in the search for new compounds [11]. Previous studies demonstrated that two bufadienolides, specifically telocinobufagin and hellebrigenin isolated from the toad *Rhinella jimi*, showed anti-*Leishmania* and anti-*T. cruzi* activity [9]. In the present study, using bioguided fractionation of the caecilian *S. annulatus* secretion, we isolated an active antiprotozoal fraction and studied its potential against *Leishmania* and *T. cruzi* parasites. By using a simple technique, namely liquid-liquid partition, an initial separation of the organic components was established, allowing the concentration of active compounds into specific fractions [22]. After testing the fractions at 300 μg/mL against parasites, the hexane and ethyl acetate fractions demonstrated activity by eliminating 100% of parasites after 24 hour of incubation. These data demonstrated that the active compounds were concentrated in the nonpolar fractions (n-hexane and ethyl acetate), suggesting a hydrophobic nature of the active compounds.

Subsequently, the active fractions from the liquid-liquid partition were separated by the RP-UPLC system, resulting in one major peak, which showed antiparasitic activity at 150 μg/mL and a similar profile in both the peak shape and retention time. Despite the high initial amount of crude secretion, the pooled active fraction yielded only 0.16% of the total mass, limiting further characterization studies in nuclear magnetic resonance (^1^H and ^13^C NMR) and mass spectrometry.

Based on the IC_50_ determination against *L. (L.) infantum*, the isolated fraction (SaFr1) was clearly more effective than the standard drug miltefosine, thus demonstrating a promising antileishmanial activity. To the best of our knowledge, the uncommon IC_50_ value found for this fraction could be included among the smallest values of natural products with antileishmanial properties against *L. (L.) infantum* promastigotes reported in the literature. Although SaFr1 was promising against the extracellular promastigotes, SaFr1 presented a lack of activity towards the intracellular amastigotes, a finding partially attributable to the lack of specific receptors in host cells, which might have contributed to blocking the penetration of SaFr1 into the intracellular macrophages [23]. Two steroids, namely telocinobufagin and hellebrigenin, isolated from the secretion of the toad *Rhinella jimi*, also demonstrated activity against *Leishmania* and *T. cruzi*, whereas similarly to our data, these secondary metabolites showed limited activity against the intracellular amastigotes [9].

Amphibian peptides have also demonstrated significant antiparasitic activity. Leite *et al.*[11] isolated six active peptides from *P. hypocondrialis* secretion (PS-1, −2, −3, −4, −5, and −6), showing activity against gram-negative and gram-positive bacteria. Only PS-4 and PS-5 demonstrated activity against *T. cruzi* trypomastigotes, with IC_50_ values of 5.1 and 4.9 μM, respectively. Brand *et al.*[12] also isolated six peptides (DShypo 1, 2, 3, 4, 6, and 7) with activity against *S. aureus*, *E. coli* and *L. (L.) amazonensis* promastigotes. Nevertheless dermaseptins 1 and 4 and phylloseptins 7 and 8, isolated from *P. nordestina*, demonstrated activity against *T. cruzi* parasites while only phylloseptin 7 presented activity against *L. (L.) infantum*[8]. Despite demonstrating antiparasitic effectiveness, SaFr1 presented high cytotoxicity *in vitro* and confirmed the lack of selectivity against intracellular amastigotes of *Leishmania infantum*. In our assays*, T. cruzi* trypomastigotes were also affected by SaFr1, but to a lesser extent than *Leishmania;* however, SaFr1 was approximately 41 times more effective than the standard drug benznidazole.

Our ultrastructural studies demonstrated that SaFr1 caused intense damage to *Leishmania* promastigotes in an incubation period of less than 60 minutes, suggesting a time-dependent autophagic process, with large vacuoles and mitochondrial swelling. Although a lethal effect was evident after 16 hours of incubation, with losses of all cytoplasmatic organelles, the SaFr1 induced no damage to plasma membrane of *Leishmania*, as confirmed by the organization of microtubules and membrane. This observation was also corroborated by our fluorimetric assay with the vital dye Sytox® green, suggesting a metabolic interference in parasites rather than a pore-forming activity. Conversely, the antimicrobial peptide, phylloseptin 7, also demonstrated a potential antileishmanial effect, but affected *Leishmania* promastigotes through the permeabilization of the plasma membrane, leading to the parasite’s death [8].

## Conclusion

Amphibian secretions represent a rich source of peptides and secondary metabolites with a wide spectrum of activities as previously reported by Toledo and Jared [10]. To the best of our knowledge, this is the first description of the antiparasitic activity found in a caecilian (*S. annulatus*) amphibian secretion, which was detected in a highly active fraction. If investigated in more detail in further studies, these data could be exploited as a tool in the search for novel biochemical targets against *Leishmania* or *T. cruzi.*

## References

[1] Alvar J, Vélez ID, Bern C, Herrero M, Desjeux P, Cano J, Jannin J, den Boer M, WHO Leishmaniasis Control Team (2012). Leishmaniasis worldwide and global estimates of its incidence. PLoS One.

[2] Croft SL, Olliaro P (2011). Leishmaniasis chemotherapy: challenges and opportunities. Clin Microbiol Infect.

[3] Ortiz MI, Suárez-Rivillas A, Molina J (2011). Behavioural responses to human skin extracts and antennal phenotypes of sylvatic first filial generation and long rearing laboratory colony *Rhodnius prolixus*. Mem Inst Oswaldo Cruz.

[4] Pereira PCM, Navarro EC (2013). Challenges and perspectives of Chagas disease: a review. J Venom Anim Toxins incl Trop Dis.

[5] Tempone AG, Sartorelli P, Mady C, Fernandes F (2007). Natural products to anti-trypanosomal drugs: an overview of new drug prototypes for American Trypanosomiasis. Cardiovasc Hematol Agents Med Chem.

[6] Camargo LB, Langoni H (2006). Impact of leishmaniasis on public health. J Venom Anim Toxins incl Trop Dis.

[7] Castilhos P, Pereira CG, Silva ALN, Napolitano DR, Oliveira F, Souza MA (2011). Effects of *Bothrops moojeni* venom on *Leishmania amazonensis* promastigote forms. J Venom Anim Toxins incl Trop Dis.

[8] Pinto EG, Pimenta DC, Antoniazzi MM, Jared C, Tempone AG (2013). Antimicrobial peptides isolated from *Phyllomedusa nordestina* (Amphibia) alter the permeability of plasma membrane of *Leishmania* and *Trypanosoma cruzi*. Exp Parasitol.

[9] Tempone AG, Pimenta DC, Lebrun I, Sartorelli P, Taniwaki NN, de-Andrade HF, Antoniazzi MM, Jared C (2008). Antileishmanial and antitrypanosomal activity of bufadienolides isolated from the toad *Rhinella jimi* parotoid macrogland secretion. Toxicon.

[10] Toledo RC, Jared C (1995). Cutaneous granular glands and amphibian venoms. Comp Biochem Physiol A Physiol.

[11] Leite JRSA, Silva LP, Rodrigues MIS, Prates MV, Brand GD, Lacava BM, Azevedo RB, Bocca AL, Albuquerque S, Bloch C (2005). Phylloseptins: a novel class of anti-bacterial and anti-protozoan peptides from the *Phyllomedusa* genus. Peptides.

[12] Brand GD, Leite JRSA, Silva LP, Albuquerque S, Prates MV, Azevedo RB, Carregaro V, Silva JS, Sá VCL, Brandão RA, Bloch C (2002). Dermaseptins from *Phyllomedusa oreades* and *Phyllomedusa distinct*. Anti-*Trypanossoma cruzi* activity without cytotoxicity to mammalian cells. J Biol Chem.

[13] Brand GD, Leite JR, de Sá Mandel SM, Mesquita DA, Silva LP, Prates MV, Barbosa EA, Vinecky F, Martins GR, Galasso JH, Kuckelhaus SA, Sampaio RN, Furtado JR, Andrade AC, Bloch C (2006). Novel dermaseptins from *Phyllomedusa hypochondrialis* (Amphibia). Biochem Biophys Res Commun.

[14] Di Marino S, Scrima M, Grimaldi M, D’Errico G, Vitiello G, Sanguinetti M, de Rosa M, Soriente A, Novellino E, D’Ursi AM (2012). Antifungal peptides at membrane interaction. Eur J Med Chem.

[15] Lorin C, Saidi H, Belaid A, Zairi A, Baleux F, Hocini H, Bélec L, Hani K, Tangy F (2005). The antimicrobial peptide dermaseptin S4 inhibits HIV-1 infectivity *in vitro*. Virology.

[16] Stauber LA, Franchino EM, Grun J (1958). An eight-day method for screening compounds against *Leishmania donovani* in the Golden Hamster. J Protozool.

[17] Kesper N, de Almeida KA, Stolf AM, Umezawa ES (2000). Immunoblot analysis of trypomastigote excreted-secreted antigens as a tool for the characterization of *Trypanosoma cruzi* strains and isolates. J Parasitol.

[18] Tada H, Shiho O, Kuroshima K, Koyama M, Tsukamoto K (1986). An improved colorimetric assay for interleukin 2. J Immunol Methods.

[19] Yardley V, Croft SL (2000). A comparison of the activities of three amphotericin B lipid formulations against experimental visceral and cutaneous leishmaniasis. Int J Antimicrob Agents.

[20] Lane JE, Ribeiro-Rodrigues R, Suarez CC, Bogitsh BJ, Jones MM, Singh PK, Carter CE (1996). *In vitro* trypanocidal activity of tetraethylthiuram disulfide and sodium diethylamine-N-carbodithioate on *Trypanosoma cruzi*. Am J Trop Med Hyg.

[21] Mangoni ML, Saugar JM, Dellisanti M, Barra D, Simmaco M, Rivas L (2005). Temporins, small antimicrobial peptides with leishmanicidal activity. J Biol Chem.

[22] Tempone AG, Martins De Oliveira C, Berlinck RG (2011). Current approaches to discover marine antileishmanial natural products. Planta Med.

[23] Tempone AG, Borborema SE, de Andrade Jr HF, de Amorim Gualda NC, Yogi A, Carvalho CS, Bachiega D, Lupo FN, Bonotto SV, Fischer DC (2005). Antiprotozoal activity of Brazilian plant extracts from isoquinoline alkaloid-producing families. Phytomedicine.

